# Exosome-mimetic vesicles derived from fibroblasts carrying matrine for wound healing

**DOI:** 10.1093/burnst/tkae015

**Published:** 2024-05-15

**Authors:** Xinyue Zhang, Jiahua Huang, Jing Zhao, Lisha Li, Fengze Miao, Tingrui Zhang, Zhongjian Chen, Xing Zhou, Zongguang Tai, Quangang Zhu

**Affiliations:** Shanghai Skin Disease Hospital, School of Medicine, Tongji University, 1278 Baode Road, Shanghai 200443, China; Shanghai Engineering Research Center for Topical Chinese Medicine, 1278 Baode Road, Shanghai 200443, China; Department of Neurology, Shanghai Public Health Clinical Center, Fudan University, 2901 Caolang Road, Shanghai 201500, China; The Central Hospital of Wuhan, Tongji Medical College, Huazhong University of Science and Technology, 26 Shengli Street, Wuhan 430014, Hubei, China; Shanghai Skin Disease Hospital, School of Medicine, Tongji University, 1278 Baode Road, Shanghai 200443, China; Shanghai Engineering Research Center for Topical Chinese Medicine, 1278 Baode Road, Shanghai 200443, China; Shanghai Skin Disease Hospital, School of Medicine, Tongji University, 1278 Baode Road, Shanghai 200443, China; Shanghai Engineering Research Center for Topical Chinese Medicine, 1278 Baode Road, Shanghai 200443, China; Shanghai Skin Disease Hospital, School of Medicine, Tongji University, 1278 Baode Road, Shanghai 200443, China; Shanghai Engineering Research Center for Topical Chinese Medicine, 1278 Baode Road, Shanghai 200443, China; Shanghai Skin Disease Hospital, School of Medicine, Tongji University, 1278 Baode Road, Shanghai 200443, China; Shanghai Engineering Research Center for Topical Chinese Medicine, 1278 Baode Road, Shanghai 200443, China; Yunnan Key Laboratory of Stem Cell and Regenerative Medicine, Science and Technology Achievement Incubation Center, Kunming Medical University, 1168 Chunrong West Road, Kunming 650500, Yunnan, China; Shanghai Skin Disease Hospital, School of Medicine, Tongji University, 1278 Baode Road, Shanghai 200443, China; Shanghai Engineering Research Center for Topical Chinese Medicine, 1278 Baode Road, Shanghai 200443, China; Shanghai Skin Disease Hospital, School of Medicine, Tongji University, 1278 Baode Road, Shanghai 200443, China; Shanghai Engineering Research Center for Topical Chinese Medicine, 1278 Baode Road, Shanghai 200443, China

**Keywords:** Wound healing, Exosome-mimetic vesicles, Matrine, Anti-inflammatory effect, Angiogenesis

## Abstract

**Background:**

Chronic skin wounds are a leading cause of hospital admissions and reduced life expectancy among older people and individuals with diabetes. Delayed wound healing is often attributed to a series of cellular abnormalities. Matrine, a well-studied component found in *Sophora flavescens*, is recognized for its anti-inflammatory effects. However, its impact on wound healing still remains uncertain. This study aims to explore the potential of matrine in promoting wound healing.

**Methods:**

In this study, we utilized gradient extrusion to produce fibroblast-derived exosome-mimetic vesicles as carriers for matrine (MHEM). MHEM were characterized using transmission electron microscopy and dynamic light scattering analysis. The therapeutic effect of MHEM in wound healing was explored *in vitro* and *in vivo*.

**Results:**

Both matrine and MHEM enhanced the cellular activity as well as the migration of fibroblasts and keratinocytes. The potent anti-inflammatory effect of matrine diluted the inflammatory response in the vicinity of wounds. Furthermore, MHEM worked together to promote angiogenesis and the expression of transforming growth factor β and collagen I. MHEM contained growth factors of fibroblasts that regulated the functions of fibroblasts, keratinocytes and monocytes, which synergistically promoted wound healing with the anti-inflammatory effect of matrine.

**Conclusions:**

MHEM showed enhanced therapeutic efficacy in the inflammatory microenvironment, for new tissue formation and angiogenesis of wound healing.

Highlights

- Fibroblasts and matrine are gradiently extruded to generate MHEM, which exhibit a good encapsulation rate of matrine.
- MHEM exert a therapeutic function throughout all stages of wound healing, thereby effectively facilitating the process of wound healing.

## Background

The skin is the largest organ in the human body. Its key functions include preventing pathogen invasion and protecting the body's internal organs [1]. Skin damage, such as pressure injuries and skin tears, is considered as an adverse event that can have a profound physical, social and psychological impact on the lives of older people. Moreover, skin wounds are the main cause of hospital admissions and amputations in patients with type 2 diabetes [2], leading to health complications and potentially shortening the lifespan of the elderly [3]. The aging population, together with the growing rates of obesity and diabetes, has led to an increase in the risks of wound healing, placing an immense burden on the healthcare system [3–5]. The general clinical wound treatment typically involves skin perfusion recovery, infection treatment and comorbidities treatment [6]. Conventional therapies may reduce symptoms adequately but have limited effectiveness for wounds with severe and recurring inflammation. [7]. Therefore, novel approaches that can efficiently and expeditiously facilitate wound healing are urgently needed.

Chronic wounds can be physiologically divided into three partially overlapping stages: inflammation, new tissue formation and remodeling [8–10]. The wound healing process has been extensively studied at the cellular level, encompassing multiple regulatory axes and signal cascades. The three stages are mainly mediated by monocytes, keratinocytes and fibroblasts [11]. Monocytes, in particular, have a crucial role in the first stage by infiltrating the wound and initiating the inflammatory responses. Their primary functions are restricting blood and fluid loss, eliminating deceased or dying cells, and preventing infection [12]. Excessive inflammation during the wound healing process can disrupt the balance of tissue regeneration, despite its important role as a natural defense mechanism against external threats. This escalated inflammation may destroy wound tissue and trigger the overproduction of extracellular matrix (ECM), thereby aggravating the formation of cicatrix [13].

Fibroblasts are indispensable in regulating the synthesis and remodeling of ECM scaffolds during wound healing. Additionally, they regulate keratinocytes in regenerating the skin barrier through autocrine/paracrine mechanisms [12, 14]. Fibroblasts exhibit remarkable activity and multiple functions to respond to injuries, maintain wound equilibrium and form new tissue.

Matrine, derived from the *Sophora flavescens* [15], is renowned for its anti-inflammatory and anti-fibrotic effects. However, its potential in the realm of wound healing through regulating the inflammatory microenvironment remains largely unexplored [16]. Recently, biotherapies based on cells, such as fibroblasts and keratinocytes, related to wound healing have received widespread attention [17]. Cell-derived nanovesicles have emerged as a promising platform for facilitating the healing of chronic wounds. Leveraging the potential of these nanovesicles, drugs can be efficiently delivered and absorbed by the wound tissue and associated cells [18, 19]. These cell-based biomaterials accelerate wound healing by promoting collagen deposition, are anti-inflammatory or promote angiogenisis [18]. However, the clinical use of cell biotechnology is considerably limited due to low productivity, challenges in large-scale production and inadequate therapeutic results [20]. Herein, we adopted a novel approach using polycarbonate membranes to perform gradient extrusion on target cells *in vitro*, which enabled us to efficiently acquire target fibroblast-derived exosome-mimetic vesicles (EM) [18]. The EM obtained in this manner have enhanced productivity and purity compared to exosomes, which could also transmit information to receptor cells and regulate receptor cell functions [21–23].

In this study, we utilized fibroblasts as the source of EM to deliver exogenous fibroblast functional molecules and matrine to the wound area. This study demonstrated the multifaceted potential of fibroblast-derived exosome-mimetic vesicles as carriers for matrine (MHEM) in promoting wound healing by accelerating inflammatory regulation through matrine and harnessing the diverse functions of fibroblast-derived exosome-mimetic nanovesicles. It also highlighted the ability of MHEM to enhance anti-inflammatory responses, angiogenesis, collagen production and epithelial closure, thereby expediting the wound healing process.

## Methods

### Materials

Dimethyl sulfoxide was obtained from Sangon Biotech (Shanghai, China). Fetal bovine serum (FBS), Dulbecco’s modified Eagle’s medium (DMEM), penicillin/streptomycin, TrypLE and phosphate-buffered saline (PBS) were from Gibco (Grand Island, CA, USA). Interferon (IFN)-γ, lipopolysaccharide (LPS), 4% paraformaldehyde fix solution, the 1,1-dioctadecyl-3,3,3,3-tetramethylindocarbocyanine perchlorate (DiI) perchlorate probe, bicinchoninic acid method (BCA) protein assay kit, reactive oxygen species (ROS) reactive oxygen assay kit and cell counting kit (CCK)-8 were purchased from Beyotime Biotech (Shanghai, China). 4′,6-Diamidino-2-phenylindole (DAPI) was from Thermo Fisher Technology (Waltham, MA, USA). Absolute ethyl alcohol, methanol and acetone were from Titan (Shanghai, China). Cell lysis buffer was from Tiangen Biotech (Beijing, China). Transwell polycarbonate cell culture inserts were purchased from Corning (Corning, NY, USA). Crystal violet staining solution and matrine standards were from Solarbio Biotech (Beijing, China). Secondary antibodies against transforming growth factor β (TGF-β) and glyceraldehyde-3-phosphate dehydrogenase (GAPDH) were purchased from Cell Signaling Technology (Boston, MA, USA). Antibodies against anti-collagen I and anti-CD31, and Masson’s trichrome staining, were from Abcam (Cambridge, UK).Radioimmunoprecipitation (RIPA) buffer,Tris-Buffered Saline Tween-20 (TBST), and color polyacrylamide gel electrophoresis (PAGE) Gel Rapid Preparation Kit were purchased from Epizyme (Shanghai, China)

### Cell lines and animals

The murine macrophage cell lines RAW264.7 and the human cell lines human skin fibroblast (HSF) and HaCat were provided by Shanghai shycbio technology CO., Ltd (Shanghai, China) and cultured in DMEM supplemented with FBS (10%) and penicillin/streptomycin (1%) at 37°C in a humidified incubator with 5% CO_2_ (Thermo, Waltham, MA, USA).

Five-week-old female BALB/c mice were procured from Shanghai JieSiJie Laboratory Animal Co., Ltd (Shanghai, China) and were maintained under specific pathogen-free conditions with a temperature of 22 ± 1°C, a relative humidity of 50 ± 1%, a light/dark cycle of 12/12 h and *ad* libitum access to standardized food and water. All animal experiments were carried out in compliance with Tongji University’s guidelines for the ethical treatment of experimental animals. The animal study was approved by the Ethics committee of Shanghai Skin Disease Hospital (No.2022–109).

### Preparation and characterization of matrine and MHEM

HSF were cultured with 20 μg mL^−1^ matrine for 4 h. Then the matrine-loaded HSF were trypsinized and resuspended in PBS containing 200 μg mL^−1^ matrine. The cell suspension underwent three rounds of extrusion through a pneumatic extruder (PhD Technology LLC, St Paul, MN, USA) equipped with 1.2, 0.6 and 0.22 μm polycarbonate filters. The obtained MHEM were dispersed in the same PBS solution as mentioned above. Matrine was initially loaded into HSF during co-incubation and subsequently encapsulated in MHEM through the repeated extrusion process.

The EM were acquired through a comparable procedure, involving the trypsinization and resuspension of HSF cells in PBS devoid of matrine, followed by the same extrusion method described above.

The protein concentration of MHEM was quantified utilizing the BCA protein assay kit. Specifically, 20 μL of the sample was added to a 96-well plate and 200 μL of BCA working solution was added to each well and incubated at 37°C for 30 min. Absorbance at 562 nm was assessed using a microplate reader (Thermo Fisher Scientific, Waltham, MA, USA).

The quantification of matrine loading in MHEM was conducted using high-performance liquid chromatography (HPLC). Briefly, the mobile phase was acetonitrile and K_2_HPO_4_ solution (0.05 mol L^−1^). The volume ratio of acetonitrile to K_2_HPO_4_ solution was 25 : 75, the flow rate was 1.0 mL min^−1^ and the detection wavelength was 220 nm. The HPLC analysis was carried out on a Waters HPLC alliance e2695 separating module (Waters, Milford, MA, USA) using a 5 μm, 4.6 × 250 mm column (Agilent, Santa Clara, CA, USA). Matrine standards were dissolved in methanol to achieve the desired concentration for analysis, which was used to create a standard curve.

The nanoparticles' zeta potential and particle size were measured with dynamic light scattering analysis using a Nano Zetasizer (Microtrac, York, PA, USA). Nanoparticle morphology was observed by transmission electron microscopy (TEM) (Hitachi, Tokyo, Japan).

### Cell viability and uptake assays

The cell viability of matrine, oxymatrine, EM and MHEM in HSF and HaCat cell lines were evaluated following established experimental procedures. HSF or HaCat were plated at a density of 5 × 10^3^ cells per well in 96-well cell culture plates and cultured overnight. Subsequently, the cells were treated for 24 h with the associated drug at concentrations ranging from 0.1 to 50 μg mL^−1^ (matrine or oxymatrine concentration) or 3 to 648 μg mL^−1^ (EM or MHEM protein concentration).

Cell viability was determined using the CCK-8 assay according to the manufacturer’s guidelines. Specifically, CCK-8 solution (10 μL) was added to each well and incubated for 0.5 h. Absorbance at 450 nm was measured using a microplate reader (Thermo Fisher Scientific, Waltham, MA, USA) and cell viability was calculated using GraphPad Prism (Version 8.3, San Diego, CA, USA).

MHEM were labeled with DiI (5 μM) orange dye for 0.5 h, then HSF were incubated with MHEM stained with DiI at protein concentrations ranging from 8 to 162 μg mL^−1^ for 4 h. Subsequently, the sample was collected, washed with PBS three times, and the cells were detected by flow cytometry using FACSCalibur (BD Biosciences, Franklin Lakes, NJ, USA).

HSF were cultured on a Millicell EZ SLIDE 8-well glass chamber (Merck, Kenilworth, NJ, USA) overnight. The medium was then replaced with fresh medium containing MHEM stained with DiI and incubated for 4 h. The cells were subsequently washed twice with PBS and incubated with Lyso-Tracker Green. Following probe removal, the cells were washed, fixed, stained with DAPI and imaged using confocal scanning microscopy (CLSM) (Leica, Wetzlar, Germany).

### Cell migration using transwell and wound healing assays

Cell migration was assessed through the utilization of a transwell experiment. Specifically, an 8.0 μm pore size transwell chamber was positioned within a 12-well plate. HSF or HaCat (1 × 10^5^ cells mL^−1^) were cultured with the corresponding drug in the upper chamber of the transwell insert, while the lower compartment contained medium (700 μL). After 24 h of incubation, the cells in the upper chamber were fixed with the crystal violet solution (0.5% in methanol) for 2 h. The transwell chamber was washed twice with PBS and any non-migratory cells were eliminated using a sterile cotton swab. The migrated cells were observed under a microscope (JASCO, Tokyo, Japan).

For the wound healing assay, HSF were cultured in a 6-well plate at a density of 2 × 10^5^ cells per well and allowed to adhere overnight. Then, a sterile 200 μL pipette tip was used to scratch a separate wound through the cells. Floating cells were gently rinsed twice with PBS while adhering cells were incubated with the corresponding drug. The progression of wound closure was documented through images captured at 0-, 12- and 24-h post-treatment using an inverted microscope (JASCO, Tokyo, Japan) at 0, 12 and 24 h of incubation.

### Tube formation

The wells in a 96-well plate were pre-coated with Matrigel. Human umbilical vein endothelial cells (HUVEC) were dispersed in DMEM containing the corresponding drug and plated in the 96-well plate at a density of 2 × 10^5^ cells per well. The cells were then cultured for 3 h and examined under a microscope (JASCO, Tokyo, Japan).

### Inflammation inhibition

RAW264.7 cells were polarized into M1 macrophages by treatment with recombinant murine IFN-γ (2 ng mL^−1^) and LPS (0.5 μg mL^−1^) for 24 h. Subsequently, the M1 macrophages were exposed to DMEM supplemented with either matrine or MHEM for 4 h. In order to visualize the presence of intracellular ROS, the cells were stained with a ROS probe, washed with PBS, fixed, and subsequently stained with DAPI. Finally, the cells were observed using CLSM.

### Animal wound model

Circular full-thickness skin samples measuring 1.5 cm in diameter were excised from the dorsal region of mice. Subsequently, 166 mM hydrogen peroxide solution was added dropwise. Then, mice were randomly divided into four groups (five mice/group): PBS group, matrine group (0.1 mL of 20 μg mL^−1^ matrine solution), EMs group (0.1 mL of EM solution) and MHEM group (matrine concentration: 20 μg mL^−1^, 0.1 mL). The therapeutic agents were subcutaneously injected at four mid-points along the wound edge, with each point receiving 25 μL of the therapeutic agents.

### Western blot

The cells were collected 24 h after culturing with treatment with specific drugs, and skin tissue was harvested from the dorsal area of mice. Both the cell and tissue samples were extracted using cold RIPA buffer containing protease inhibitor cocktail (NCM Biotech). Subsequently, the samples were separated by 15% sodium dodecyl sulfate -polyacrylamide gel electrophoresis (SDS-PAGE) and transferred onto polyvinylidene difluoride membranes (Millipore, Mississauga, Canada). Following blocking of the membranes using TBST with 5% skim milk for 1 h, membranes were incubated overnight at 4°C with primary antibodies targeting GAPDH, type I collagen (COL-I) and TGF-β in solution. After being washed extensively, the samples were incubated with anti-rabbit secondary antibodies at room temperature for 1 h. Subsequently, the proteins on the membranes were detected using a chemiluminescence kit (NCM Biotech, Suzhou, Jiangsu province, China) and visualized with the ChemiDoc MP Imaging System (BioRad, Hercules, CA, USA).

### Histological analysis

The newly formed skin tissues extracted from the dorsal region of the mice were promptly fixed upon removal. Subsequently, the tissues underwent dehydration and were preserved in 4% paraformaldehyde. Following this, a slicer was employed to section the tissues for further experiments including hematoxylin–eosin (H&E) staining, Masson trichrome staining and immunohistochemical staining. Specifically, skin tissues were embedded in paraffin, cut into slices and stained with H&E and Masson trichrome to evaluate the histological changes under a microscope (H550S, Nikon, Japan). Immunohistochemical analysis was conducted by using CD31-specific antibodies. After the color reaction, slices were used to observed the proportion of positive cells.

The major organs (heart, liver, spleen, lung, kidney) were harvested from mice following treatment with PBS, matrine, EM and MHEM. As previously described, the major organs were fixed, sectioned and subjected to H&E staining as outlined previously, with subsequent examination of the tissue slices under a microscope (H550S, Nikon, Japan).

### Statistical analysis

The data was expressed as mean ± S.D. and analyzed by GraphPad Prism software. Statistical significance was assessed through one-way analysis of variance (ANOVA), two-way ANOVA and student's t-test. A *P* value of <0.05 was marked as ‘^*^’, a *P* value of <0.01 was marked as ‘^*^^*^’ and a *P* value of <0.001 was marked as ‘^*^^*^^*^’. Tukey’s multiple comparisons test was used for *post hoc* testing after ANOVA.

## Results

### Preparation and characterization of MHEM

**Figure 1:**
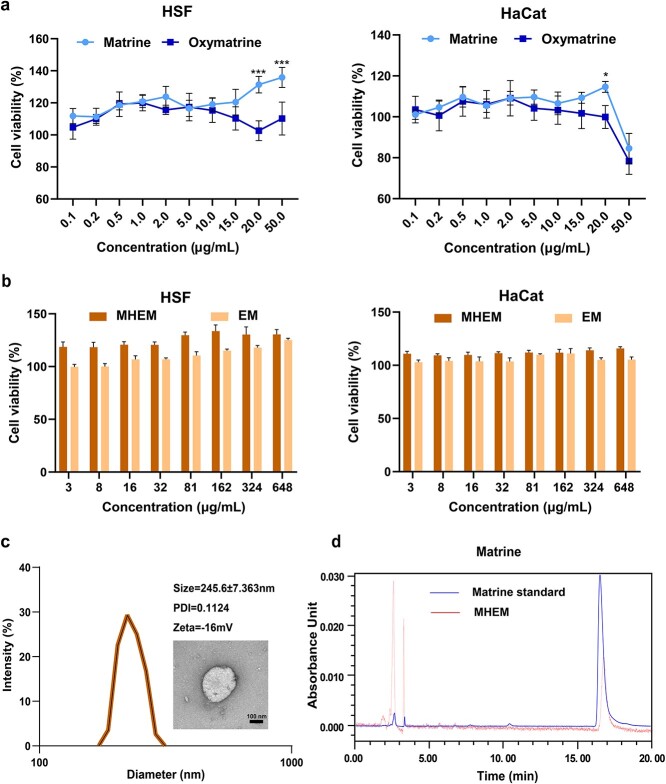
Characterization of MHEM. (**a**) Cell viability of HSF and HaCat treated with different concentrations of matrine and oxymatrine. (**b**) Cell viability of HSF and HaCat treated with a different protein concentrations of MHEM and EM (matrine concentration: 20 μg mL^−1^). (**c**) Particle size distribution, polydispersity index, zeta potential and TEM image of MHEM. Scale bar: 100 nm (n = 3). (**d**) Determination of matrine in MHEM by HPLC. Data are presented as mean ± S.D. ^*^p < 0.05; ^*^^*^^*^p < 0.001. *MHEM* fibroblast-derived exosome-mimetic vesicles as carriers for matrine, *EM* fibroblast-derived exosome-mimetic vesicles, *HPLC* high-performance liquid chromatography, *TEM* transmission electron microscopy, *HSF* human skin fibroblast, *HaCat* human immortalized epidermal cells, *PDI* polydispersity index

Matrine and oxymatrine can interconvert *in vivo*, but they exhibit diverse effects on the human body [24]. To determine the ideal form and the best concentration that interacts with cells, we conducted a coincubation experiment involving HSF and HaCat. The results revealed that matrine positively influenced cell viability in both cell types, with 20 μg mL^−1^ of matrine showing the most significant effect in enhancing cell viability (Figure 1a). Matrine at a concentration of 20 μg mL^−1^ had a more pronounced impact on the viability of both types of cells compared to oxymatrine.

Subsequently, an examination was conducted to assess the effects of EM containing different protein concentrations on cell proliferation. The results, depicted in Figure 1b, indicated a significant increase in cell proliferation when the protein concentration reached 162 μg mL^−1^. Furthermore, the use of MHEM demonstrated a more pronounced impact on promoting cell proliferation. The decrease in fibroblast viability was identified as a potential factor in delayed wound healing [25]. Thus, enhancing the function of fibroblasts can further promote wound healing. The optimal working solution for HSF and HaCat was determined to be a medium containing 162 μg mL^−1^ protein and 20 μg mL^−1^ matrine.

The particle size of the MHEM, as measured by dynamic light scattering, was found to be ~245 nm, with a polydispersity index (PDI) of 0.11, indicating a uniform particle size distribution of MHEM. The zeta potential of the MHEM was measured to be −16 mV, a value comparable to that of the cell membrane. TEM imaging indicated that the MHEM displayed a vacuolar overall shape, with a membrane-like structure enveloping the outer layer (Figure 1c). Subsequently, HPLC analysis was employed to quantify the matrine loading within the MHEM. The vesicles exhibited a peak concurrent with matrine, and the encapsulation efficiency was found to be ~77 ± 2.59% (Figure 1d).

### Internalized MHEM promote HSF and HaCat migration

**Figure 2:**
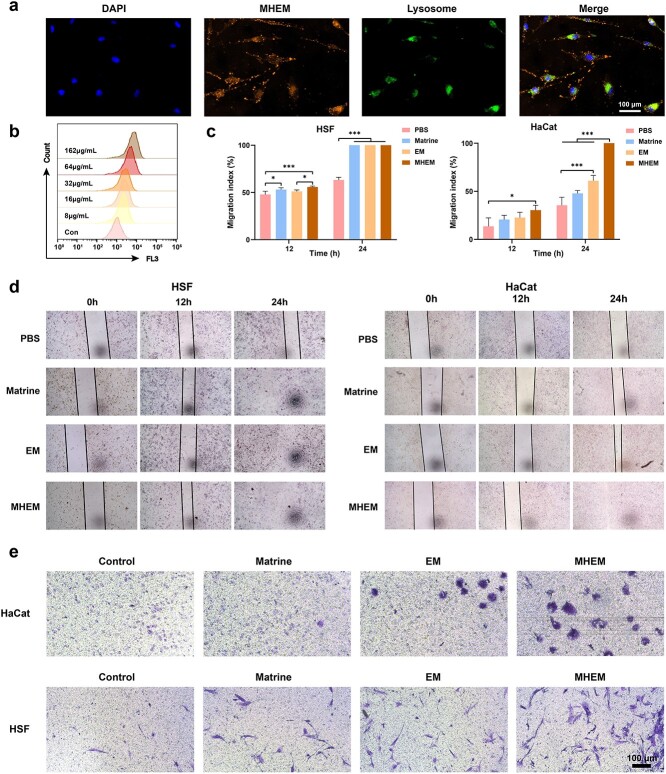
Role of MHEM in cell uptake and promoting cell migration *in vitro.* (**a**) CLSM images of DiI-labeled MHEM after coincubation with HSF (scale bar: 100 μm). (**b**) Cellular uptake of MHEM detected by flow cytometry assay on HSF. (**c**) Statistical analysis of the migration ability of HSF and HaCat measured by scratch assay (n = 3). (**d**, **e**) Migration ability of HSF and HaCat after treatment with matrine, EM and MHEM measured by wound healing assay (d) and transwell assay (e). Scale bar: 100 μm. Data are presented as mean ± S.D. ^*^p < 0.05; ^*^^*^^*^p < 0.001. *MHEM* fibroblast-derived exosome-mimetic vesicles as carriers for matrine, *EM* fibroblast-derived exosome-mimetic vesicles, *HSF* human skinfibroblast, *HaCat* human immortalized epidermal cells, *CLSM* confocal scanning microscopy, *Con* control, *DiI* 1,1-dioctadecyl-3,3,3,3-tetramethylindocarbocyanine perchlorat

MHEM were labelled with DiI, while the lysosomes of HSF were stained in green fluorescence (Figure 2a). Subsequently, the uptake and intracellular localization of MHEM in HSF were observed through CLSM. Interestingly, following a 4-h period, MHEM were found to accumulate in the cytoplasm and cell membrane of HSF, indicating successful uptake and processing by the cells [26–28]. It is noteworthy that a portion of the MHEM did not co-localize with the lysosomes, suggesting evasion of lysosomal degradation. Furthermore, we evaluated MHEM uptake by HSF using flow cytometry. Our findings revealed a clear correlation between the protein concentration of vesicles and their uptake by HSF. When the protein concentration reached 162 μg mL^−1^, HSF exhibited significantly enhanced absorption efficiency (Figure 2b).

The migration of keratinocytes and fibroblasts plays a crucial role in wound healing, specifically in wound contraction and re-epithelization [12, 29, 30]. In this study, we investigated the impact of MHEM on the migration of HSF and HaCat using scratch experiments (Figure 2c, d). After 12 h of coincubation, the presence of matrine significantly promoted the migration of HSF, and MHEM exhibited a remarkably stronger ability to enhance HSF migration compared to EM. After 24 h coincubation, the scratches (wound created in the wound healing assay) of HSF in matrine, EM and MHEM groups were essentially healed, suggesting the remarkable potential of matrine, EM and MHEM in promoting HSF migration.

The results indicated that HaCat internalizing MHEM demonstrated enhanced migration capacity compared to other groups. Although no significant difference was observed between the matrine group and the PBS group in terms of promoting the migration of HaCat, MHEM exhibited more pronounced effect in promoting HaCat migration than the EM. This finding suggested that the migration ability of HaCat was enhanced through a synergistic mechanism of matrine and EM, further underlining the potential of MHEM as a therapeutic option for wound healing.

Furthermore, the migratory capacity of HSF and HaCat co-cultured with MHEM was confirmed through the transwell assay (Figure 2e). The results demonstrated that MHEM exerted the strongest effect in promoting the migration of HSF and HaCat, while matrine and EM acted synergistically to enhance the migration of HSF and HaCat.

### MHEM inhibit ROS, promote angiogenesis and increase the expression of TGF-β and COL-I *in vitro*

**Figure 3:**
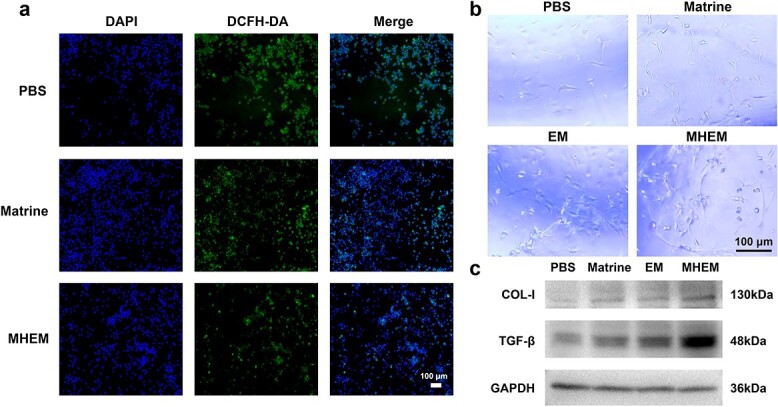
MHEM inhibit the production of ROS in M1 macrophages, and promote HUVEC tube formation and the expression of wound healing-related proteins in HSF. (**a**) Inhibitory effect of MHEM on ROS generation in M1 macrophages (scale bar: 100 μm). (**b**) Tube formation assay of HUVECs (scale bar: 100 μm). (**c**) Western blot analysis of protein levels of TGF-β and COL-I in HSF. *MHEM* fibroblast-derived exosome-mimetic vesicles as carriers for matrine, *EM* fibroblast-derived exosome-mimetic vesicles, *HSF* human skinfibroblast, *HaCat* human immortalized epidermal cells, *ROS* reactive oxygen species, *DAPI* 4',6-diamidino-2-phenylindole, *DCFH-DA* 2',7'-dichlorodihydrofluorescein diacetate, *HUVEC* Human umbilical vein endothelial cells, *COL-I* collagen I, *TGF-β* transforming growth factor β, *GAPDH* glyceraldehyde-3-phosphate dehydrogenase

We induced polarization of macrophages towards the M1 phenotype to mimic the inflammatory environment of a wound. M1 macrophages, known for their pro-inflammatory properties, demonstrate elevated levels of intracellular ROS. Interestingly, the fluorescence intensity emitted by ROS probes within M1 macrophages was significantly higher compared to other treated macrophages (Figure 3a). After the coincubation of M1 macrophages with matrine, we observed a decrease in the fluorescence intensity of the ROS probe (Figure 3a). Furthermore, when M1 macrophages were coincubated with MHEM, the ROS level in these macrophages was significantly reduced. MHEM exhibited stronger ROS inhibitory effect than matrine, possibly due to the biocompatibility and the nanoscale size of MHEM, facilitating enhanced uptake of MHEM by macrophages [31]. Consequently, MHEM promoted resolution of the inflammatory response, accelerating the transition to the second phase of wound healing.

In this study, we used the HUVEC tube formation assay to investigate the potential of MHEM in promoting angiogenesis. Our findings revealed that upon coincubation with EM or matrine for 4 h, the distinct formation of the HUVEC tube was observed (Figure 3b). Notably, the tube structure and morphology were more pronounced when HUVEC were treated with MHEM, suggesting a superior angiogenic effect compared to EM or matrine.

Therefore, we conducted an analysis of growth factors and proteins related to wound healing, specifically focusing on COL-I and TGF-β due to their significant physiologically and pathological roles in this process [32]. In this study, western blot assay was used to assess the protein levels of HSF following coincubation with MHEM. As shown in Figure 3c, MHEM enhanced the expression of COL-I and TGF-β in HSF, indicating that MHEM can potentially promote the proliferation of granulation tissue to accelerate wound healing.

### Ability of MHEM to promote wound healing *in vivo*

**Figure 4:**
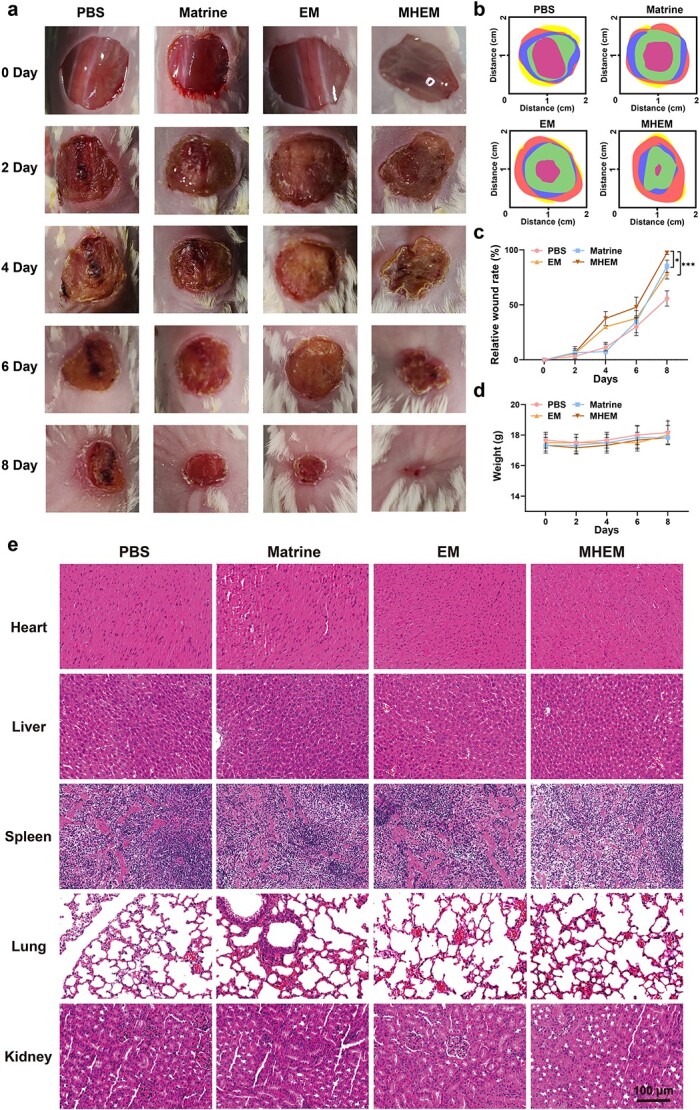
Assessment of wound healing efficiency of MHEM *in vivo*. (**a**) Macroscopic images of wounds at different time points. (**b**) Wound closure traces for each group of mice in 8 days. (**c**) Quantitative size of the wound area in different groups. (**d**) Body weight of mice over a treatment period of 10 days. (**e**) H&E staining of major organs of mice subcutaneously injected with the indicated treatments (Scale bar: 100 μm). Data are presented as mean ± S.D. ^*^p < 0.05; ^*^^*^^*^p < 0.001. *MHEM* fibroblast-derived exosome-mimetic vesicles as carriers for matrine, *EM* fibroblast-derived exosome-mimetic vesicles, *H&E* hematoxylin–eosin

To assess the efficacy of MHEM in wound healing *in vivo*, we created full-thickness excised wounds on the backs of the mice, with a diameter of ~1.5 cm. Hydrogen peroxide was dripped onto the wound to simulate the aggressive inflammatory microenvironment. Subsequently, MHEM were subcutaneously injected into the edge of the wound, starting from the first day of modeling, which was then repeated every other day for a total of three times. The healing progression of the wounds in the mice is illustrated in Figure 4a, b. Compared to the PBS group, both MHEM and EM groups exhibited gradual healing from day 4 onwards. Furthermore, the matrine promoted wound healing from day 6 onwards. This finding suggested that suppressed inflammatory response accelerated the second or third stage of wound healing (Figure 4c).

On day 8, the mice treated with matrine and EM exhibited a notable reduction in wound size, indicating advancement to the proliferative stage of wound healing [33]. Remarkably, wounds in the MHEM group healed completely by day 8, indicating that matrine and EM synergistically promoted wound healing. Meanwhile, the biosafety of vesicles was confirmed as the subcutaneous injection of vesicles did not induce any significant changes in mouse body weight (Figure 4d). No discernible inflammation or damage to the major organs was observed in any instance, indicating minimal toxic side effects and the biosafety of EM and MHEM during the course of treatment (Figure 4e).

### MHEM promote wound healing through enhanced angiogenesis and expression of TGF-β and COL-I *in vivo*

**Figure 5:**
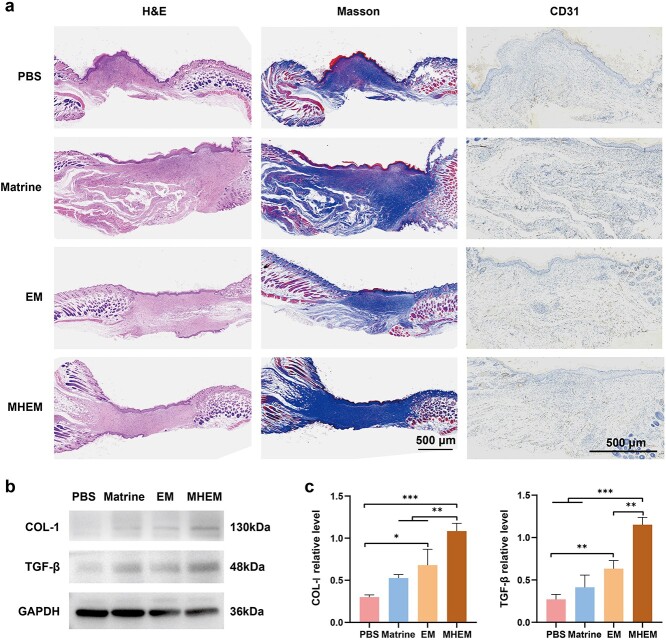
Specific performance of MHEM promoting wound healing *in vivo*. (**a**) H&E, Masson and CD31 staining images of wound tissues (scale bar: 500 μm). (**b**) Western blot of wounds and (**c**) statistical analysis (n = 3). Data are presented as mean ± S.D. ^*^p < 0.05; ^*^^*^p < 0.01; ^*^^*^^*^p < 0.001. *MHEM* fibroblast-derived exosome-mimetic vesicles as carriers for matrine, *EM* fibroblast-derived exosome-mimetic vesicles, *COL-I* collagen I, *TGF-β* transforminggrowth factor β, *GAPDH* glyceraldehyde-3-phosphate dehydrogenase, *H&E* hematoxylin–eosin

H&E staining was used for histological assessment of mouse wounds. As shown in Figure 5a, the wounds treated with PBS exhibited persistent subcutaneous bleeding and inflammation characteristics on day 8. Conversely, the matrine, EM and MHEM groups had formed new skin and blood vessels. However, the H&E images demonstrated that the wounds of the MHEM group exhibited the highest wound healing efficiency, with the mature formation of epidermal and dermal tissues compared to other groups. Figure 5a showed that matrine, EM and MHEM could promote collagen deposition, and the content of collagen deposition in the MHEM group was higher than in other groups. These findings suggested that cell-derived nanovesicles further enhanced the uptake and utilization of matrine by cells, thereby synergically promoting collagen deposition.

CD31, a platelet endothelial cell adhesion molecule recognized as a biomarker of vascular endothelial cells [34], has a crucial role in angiogenesis and wound healing [35]. Along with *in vitro* findings, the *in vivo* results of histochemical staining suggested that both matrine and EM could stimulate CD31 expression, indicating their potential to induce angiogenesis. Western blot results showed that the expression levels of COL-I and TGF-β were significantly higher in the matrine and the EM groups than in the PBS group (Figure 5b, c). Also, MHEM might enhance wound healing by promoting the formation of epidermal, dermal and vascular tissues, increasing collagen deposition and upregulating the expression of COL-I and TGF-β.

## Discussion

In the field of wound healing, commercially available biomaterials are commonly used with the aim of alleviating fluid exudation and scarring. However, the present focus of research on advanced biomaterials for wound healing is primarily centered around effectively addressing inflammation [36]. One such promising biomaterial is MHEM, which has been found to promote wound healing through multiple mechanisms including anti-inflammatory effects, collagen deposition, tissue regeneration and angiogenesis. MHEM were made up of membranes. These nanovesicles possessed adhesion proteins homologous to fibroblasts, thus allowing targeted homologous binding to fibroblasts [37]. Upon fusion with the cell, MHEM released their contents into the cell, thus effecting cell functions [21, 25].

Wounds are commonly categorized into two categories, namely acute and chronic. Acute wound healing involves a progression through three stages: inflammation, repair and remodeling. However, if acute wounds fail to transition into the repair and remodeling stage, they become chronic wounds. Chronic wounds, which are particularly challenging to heal due to their prolonged and more severe inflammation compared to acute wounds, attract a substantial influx of pro-inflammatory macrophages in the vicinity of the wounds [38]. Pro-inflammatory macrophages are known to release a substantial amount of ROS, leading to oxidative stress. Also, increased level of ROS contribute to the necrosis of numerous skin cells. Therefore, inhibiting the exaggerated inflammatory response is crucial in safeguarding the wound tissue against excessive oxidative stress [13, 39–41]. MHEM effectively alleviated oxidative stress by inhibiting the pro-inflammatory function of M1 macrophages. Persistent and intense inflammation may exacerbate scar formation. This result also suggested the potential of matrine to reduce cicatrix formation and promote the healing of immature wound tissue through inflammation inhibition [42].

Following the resolution of inflammation, the subsequent phase of wound healing begins, characterized by the infiltration of nascent capillaries into the wound tissue, ultimately forming a microvascular network [12, 32]. The complex network of blood vessels that develops through angiogenesis, which plays a crucial role in accelerating tissue regeneration by facilitating the transport of oxygen and nutrients to wound sites, thereby accelerating tissue regeneration. Therefore, angiogenesis is a significant marker of the reparation and regeneration of the skin tissue. The formation of HUVEC tube assay was used to analyze the angiogenesis process* in vitro*, MHEM effectively encouraged HUVEC tube formation, thus highlighting their promising potential in promoting angiogenesis.

Furthermore, MHEM could enhance the secretion of TGF-β and COL-I by HSFs. TGF-β, a transforming growth factor, regulates various cellular functions during the wound healing process, including epithelialization, fibroblast migration, collagen synthesis and maturation, and ECM deposition [43], which is one of the markers of the second stage of wound healing. Additionally, fibroblast-produced collagen, particularly COL-I, contributes to tissue strength enhancement [42] and facilitates pulling the wound tissue edges towards the center. The increase of COL-I and enhancement of tissue strength represent the ultimate phases of wound healing [12].

In summary, MHEM possessed the capacity to impede inflammation, foster angiogenesis, and stimulate the synthesis of TGF-β and COL-I. Consequently, MHEM facilitated wound healing throughout all three stages of the process. Therefore, the potential of MHEM in enhancing wound healing warrants further exploration and development.

## Conclusions

Exosome mimetics derived from human skin fibroblasts containing matrine, i.e. MHEM, improved the compatibility of matrine while preserving the wound healing-related cytokines and proteins produced by HSF cells, such as TGF-β and COL-I. MHEM promoted wound healing through their anti-inflammatory properties, promotion of angiogenesis, and increased secretion of cytokines and proteins. The combination of matrine and HSF-derived exosome mimetics exhibited a synergistic effect, playing a multifaceted role in various stages of wound healing.

## Data Availability

The data that support the findings of this study are available within this article or from the corresponding author upon reasonable request.
